# Listeriosis, Taiwan, 1996–2008

**DOI:** 10.3201/eid1709.110093

**Published:** 2011-09

**Authors:** Yu-Tsung Huang, Chun-Hsing Liao, Chia-Jui Yang, Lee-Jene Teng, Jin-Town Wang, Po-Ren Hsueh

**Affiliations:** Author affiliations: National Taiwan University Hospital, Taipei, Taiwan (Y.-T. Huang, L.-J. Teng, J.-T. Wang, P.-R. Hsueh);; Far Eastern Memorial Hospital 4, New Taipei City, Taiwan (Y.-T. Huang, C.-H. Liao, C.-J. Yang)

**Keywords:** listeriosis, Listeria, bacteria, clustering, zoonoses, Taiwan, dispatch

## Abstract

During 1996–2008, a total of 48 patients with listeriosis were identified at a Taiwan hospital. Average annual incidence increased from 0.029 to 0.118 cases per 1,000 admissions before and after January 2005. Serotype 1/2b predominated; serotype 4b emerged since 2004. Food monitoring and disease surveillance systems could help control listeriosis in Taiwan.

*Listeria monocytogenes* is a gram-positive bacillus that exists in contaminated food and animal products (*1*). Certain serotypes (1/2a, 1/2b, 1/2c, and 4b) are associated with most human diseases and have caused several outbreaks (*1*). Clinical features of human listeriosis include self-limiting gastroenteritis in outbreak cases, spontaneous abortion in pregnant women, and severe infections (sepsis and meningitis) in immunocompromised persons and in elderly persons. In the latter, the case-fatality rate is 20%–30% (*1*). The incidence of nonpregnancy-associated listeriosis has increased recently in Europe despite strict food regulations (*2**,**3*).

In Taiwan, unlike in other Asian countries, human listeriosis was rarely reported, although *L. monocytogenes* has been isolated from local farm products (*4**–**6*). Recent surveillance of neonatal listeriosis in Taiwan identified an increase in cases after 2000 (*5*). However, there are a paucity of data on serotyping and molecular epidemiology of human listeriosis in Taiwan because the disease is not nationally notifiable (*7*). We investigated nonpregnancy-associated listeriosis in adults, as well as serotyping and genetic relatedness for all isolates identified in our hospital.

## The Study

We reviewed the medical records of patients who had *L. monocytogenes* isolated from blood and body fluids from sterile sites during 1996–2008 at the National Taiwan University Hospital (NTUH), a 2,500-bed hospital in Taiwan. Demographic and clinical data of nonpregnant adults with listeriosis were retrieved for further analysis. We evaluated disease severity using modified Acute Physiology And Chronic Health Evaluation II scores (*8*). Incidence of nontyphiodal *Salmonella* bacteremia (NTSB) during 2000–2008 in NTUH was calculated for trend comparison of the 2 foodborne illnesses. Only 1 episode was calculated during the same admission for NTSB to avoid the influence of repetitive bacteremia.

Isolates from patients with fetomaternal listeriosis (i.e., paired isolates from mother and neonate who had listeriosis) were considered to be the same and only 1 of them was analyzed. All isolates were analyzed for their serotype by PCR as described, and genetic relatedness was evaluated by pulsed-field gel electrophoresis (PFGE) by using PulseNet standardized protocols and 2 restriction enzymes (*Asc*I and *Apa*I) (*2**,**9*). Strains were considered to be of the same cluster if their bands had indistinguishable restriction patterns by both enzymes. Strains with PFGE patterns with >80% similarity by *Asc*I and *Apa*I profiles were considered to be closely related. A forward stepwise model with a p value of 0.1 was used, and p<0.05 was considered statistically significant in the multivariate Cox proportional hazards model.

**Figure 1:**
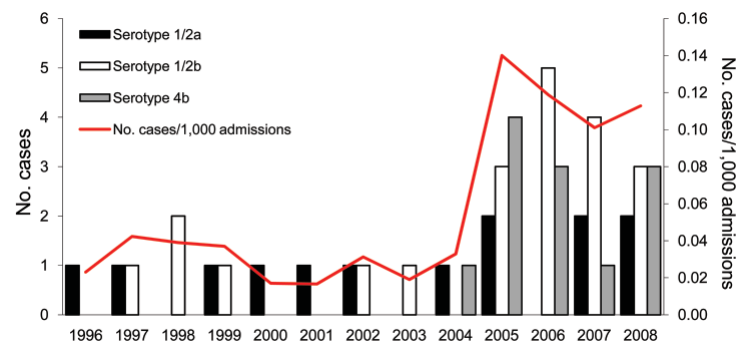
Incidence (cases per 1,000 admissions) of human listeriosis and serotype distribution of all isolates, National Taiwan University Hospital, Taipei, Taiwan, 1996–2008. Forty-six isolates were available for analysis, including 2 serotype 4b isolates from fetomaternal transmission in 2005 and 2006 and 1 serotype 4b from a pediatric patient (2005). Isolates from fetomaternal transmission were considered to be the same.

**Figure 2:**
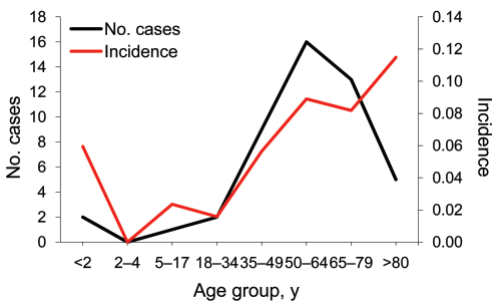
Patient distribution and incidence of human listeriosis, by age group, National Taiwan University Hospital, Taipei, Taiwan, 1996–2008.

During the study period, listeriosis was diagnosed in 48 patients, and 46 nonduplicated isolates were obtained for further microbiological analysis. Average annual incidence increased from 0.0287 cases per 1,000 admissions during 1996–2004 to 0.118 cases per 1,000 admissions during 2005–2008 (Figure 1). The increase in annual incidence of listeriosis was significantly correlated with years (p = 0.0045). The average annual incidences of NTSB were 1.189 and 1.118 per 1,000 admissions during 2000–2004 and 2005–2008, respectively; it was not significantly correlated with years (p = 0.50). Age-specific incidence of listeriosis increased at both extremes of age, but especially among patients >80 years (Figure 2). All of the patients with listeriosis lived in northern Taiwan, and no obvious geographic correlation was observed between listeriosis patients in each year.

We reviewed 43 cases of listeriosis in nonpregnant adults (Table). All 43 patients had underlying predisposing conditions, and 18 (42%) were >65 years of age. Of the 30 patients with malignancies, 23 (77%) developed listeriosis within 4 weeks after receiving chemotherapy.

**Table:** Demographic data for 43 nonpregnant adults with listeriosis, National Taiwan University Hospital, Taipei, Taiwan, 1996–2008*

Characteristic	No. (%) patients, n = 43
Sex	
M	29 (67)
F	14 (33)
Underlying condition	
Steroid use >0.5 mg/kg/day	19 (44)
Diabetes mellitus	11 (26)
Surgery in previous 2 mo	10 (23)
Peptic ulcer disease with or without bleeding	9 (21)
Liver cirrhosis†	9 (21)
Renal insufficiency‡	8 (19)
Alcoholism	4 (9)
Ulcerative colitis	2 (5)
Antiphospholipid syndrome	1 (2)
Hematologic malignancy	15 (35)
Multiple myeloma	5 (12)
Non-Hodgkin lymphoma§	5 (12)
Leukemia	4 (9)
Myelodysplastic syndrome	1 (2)
Solid cancer	19 (44)
Lung cancer	4 (9)
Malignancy of unknown primary hepatocellular carcinoma	3 (7)
Gastric cancer§	2 (5)
Colorectal cancer§	2 (5)
Bladder cancer§	2 (5)
Ovarian cancer	2 (5)
Chemotherapy within 4 weeks	23 (53)
Diagnosis¶	
Bacteremia	29 (67)
Meningitis/meningoencephalitis	8 (19)
Spontaneous bacterial peritonitis	4 (9)
Brain abscess	2 (5)
Crude mortality rate	
7 d	11 (26)
14 d	12 (28)

*Mean age, y, ± SD: 62.12 ± 15.10. †Including 4 patients with hepatic decompensation. ‡Including 3 patients with end-stage renal insufficiency who were receiving hemodialysis. §One female patient had coexisting non-Hodgkin lymphoma and colon cancer; 1 male patient had coexisting non-Hodgkin lymphoma, gastric cancer, and bladder cancer. ¶Initial modified Acute Physiology And Chronic Health Evaluation II score, mean ± SD: 18.8 ± 7.3.

Among the 46 isolates, serotype 1/2b was identified most frequently (46%), followed by 1/2a (28%) and 4b (26%). No serotype 1/2c was detected by PCR. Serotype 4b was noted beginning in 2004. PFGE results showed no shared pulsotypes. All 46 isolates were susceptible to ampicillin, ertapenem, meropenem, and vancomycin; 3 isolates were intermediately susceptible to trimethoprim/sulfamethoxazole; and 4 isolates were nonsusceptible to linezolid (*10*).

The all-cause death rate at day 14 of hospitalization was 28%. Sixteen (37%) patients received cephalosporin alone as initial treatment regimens and empirical treatment was effective for only 14 (33%). The presence of solid-organ malignancies was a significant negative prognostic factor for 14-day mortality in the univariate analysis (95% confidence interval [CI] 1.165–16.694; p = 0.029) but not in the multivariate analysis. The results of the multivariate analysis for 14-day mortality showed that hepatic decompensation at disease onset was a significant negative prognostic factor (hazard ratio 12.02, 95% CI 1.842–78.470; p = 0.009) and that the use of effective antimicrobial drugs after culture results were reported was a significant positive prognostic factor (hazard ratio 0.014, 95% CI 0.002–0.131; p<0.001). Log-rank tests performed to compare the difference in survival between patient groups for the 2 variables also had the same results (p<0.001).

## Conclusions

We observed an upsurge of listeriosis beginning in 2005 in NTUH. The increase might not be attributable to common-source outbreaks because no clustering was detected. The annual incidence of listeriosis has been on the rise in Europe since 2000 (*2**,**3**,**11*). The reason is not clear because the increase could not be attributed to outbreak clusters and no increase in pregnancy-related listeriosis was observed (*2**,**3*).

*L. monocytogenes* isolates are not uncommon in domestic food products in Taiwan. Wong et al. found that *L. monocytogenes* was isolated in >50% of pork samples and chicken carcasses (*6*). Semiready foods (dumplings and meatballs) and frozen dim sum examined also carried the pathogen (34.0% and 4.4%, respectively), and >60% of the isolates were serotype 1 or 4 (*6*). If served undercooked, these foods could be potential transmission sources. Taiwan currently has no strict regulatory policy regarding listeriosis in the food industry and no disease surveillance system.

In France, serotype 4b was the predominant serotype (42%–56%), whereas serotype 1/2b was more common (46%) in our study (*2*). Detection of resistance is not routinely performed in most laboratories. In our study, none of the isolates were resistant to the tested agents, except for 4 isolates, which were resistant to linezolid. The clinical efficacy of these new agents should be carefully evaluated.

The contributions of disease severity and antimicrobial drug treatment are difficult to evaluate in population-based studies (*12*). Brouwer et al. reported that up to 30% of adults with *L. monocytogenes* meningitis did not receive initial adequate antimicrobial drug therapy, and they found no association between that variable and outcome (*13*). A high proportion (37%) of patients in our study also received inadequate antimicrobial drug therapy initially. Initial disease severity and initial adequate antimicrobial drug therapy was not associated with overall mortality. We found that mortality was related to hepatic decompensation and effective antimicrobial drug therapy after culture results were reported. However, further studies comprising larger patient populations are necessary to confirm our findings.

Our data were based on patients in a single hospital. Therefore, the incidence of and risk factors for human listeriosis in Taiwan could not be determined precisely, and potential outbreaks might have been overlooked. The retrospective design of our study limited the possibility of identifying the potential vehicles and disease-acquiring behaviors.

An increase of listeriosis was noted since 2005 in our hospital, and all of the affected patients had predisposing factors that hampered their immunity. Dietary education and food management information should be provided to high-risk groups in Taiwan. Food monitoring and human disease surveillance systems need to be established in Taiwan to control this potentially fatal foodborne disease.
